# Looking Beyond Thyroid Malignancy: An Unusual Case of Dyshormonogenetic Goiter in Rural India

**DOI:** 10.7759/cureus.68139

**Published:** 2024-08-29

**Authors:** Anushka Dekhne, Apurva Popat, Arun Chopwad

**Affiliations:** 1 Internal Medicine, American University of Antigua, Antigua, ATG; 2 Internal Medicine, Marshfield Clinic Health System, Marshfield, USA; 3 Pathology, Ashwini Sahakari Rugnalaya Ani Sanshodhan Kendra, Solapur, IND

**Keywords:** goiter, dyshormonogenetic goiter, pediatric endocrinology, microscopy, surgical pathology, malignancy

## Abstract

Dyshormonogenetic goiter (DG) is a rare cause of congenital hypothyroidism (CH) occurring due to the lack of enzymes necessary for thyroid hormone synthesis. If left untreated, it impairs hormone production leading to developmental and metabolic complications. Morphologically, it is characterized by architectural and cellular pleomorphism that may mimic thyroid malignancy causing difficulties in diagnosis. Thus, accurate histopathological evaluation is crucial in distinguishing DG from malignancy. We report a case of a 13-year-old female diagnosed with hypothyroidism at the age of six. Over time, she exhibited slow development of a multinodular goiter and began experiencing dyspnea in the supine position. Ultrasonography confirmed an enlarged thyroid gland with solid hypoechoic nodules devoid of calcifications, so a total thyroidectomy was performed. Gross examination revealed that the gland was notably enlarged with a grey-tan nodular appearance with few cystic hemorrhagic areas and had a firm rubbery texture. Microscopy identified microfollicular cells with significant hyperplasia and cytologic atypia along with scant colloid, indicative of DG. Histopathological literature has been essential to prevent overdiagnosis of malignancy. Additionally, the authors suggest that it is crucial to include DG in the differential diagnosis when evaluating potential causes of CH.

## Introduction

In the Indian pediatric age group, dyshormonogenetic goiter (DG) is one of the common causes of congenital hypothyroidism (CH); however, its literature is largely an extrapolation of CH, and the risk categorization remains speculative. Further, the literature on pediatric DG remains very sparse in the Indian sub-continent [1].

DG is the second most common cause of CH accounting for 10%-15% of the cases after thyroid dysgenesis and is often underreported in these regions [2]. The primary causes for dyshormonogenesis include impaired organification of iodide and faulty secretion of thyroglobulin [3]. Despite the underlying quantitative deficiency of thyroid hormone associated with DG, patients may present as clinically euthyroid [2]. The substantial hyperplasia often observed in DG may lead less experienced clinicians to mistakenly suspect malignancy due to the pronounced changes in the gland's architecture [4-6]. Considering the potential morbidity and risk to psychomotor development associated with untreated/mistreated DG, early detection and management are critical to prevent neurodevelopmental impairment in children [7].

In this report, we discuss a unique case of DG in a 13-year-old girl showing signs from as early as three years old, in a rural area in India. It took the authors nearly a decade to confirm this diagnosis, highlighting the challenges in identifying DG in areas prone to iodine deficiency disorders.

## Case presentation

The patient was a 13-year-old girl, hailing from a rural region in India. She presented with complaints of difficulty in breathing while asleep and a visible swelling in the neck region. The patient was the only offspring with no maternal history of hypothyroidism or intake of goitrogenic substances during pregnancy. Her parents revealed that the neck swelling was observed since the patient was three years old, and gradually enlarging over the years to its current size. It was communicated that her developmental milestones were also delayed. Upon further questioning, it was disclosed that the local healthcare providers diagnosed her with CH and the patient had been receiving Eltroxin (levothyroxine) 50 mcg for the last seven years.

On physical examination, she appeared clinically hypothyroid exhibiting a short stature, poor muscle tone, and dry skin. The thyroid was soft to firm with an irregular surface with no cervical lymphadenopathy, bruit, or retrosternal extension. The ultrasound imaging revealed an enlarged thyroid gland with solid hypoechoic nodules and an isthmus which was readily visualized. No calcifications were seen, and involvement of any blood vessels was not observed.

The laboratory evaluation revealed elevated levels of thyroid-stimulating hormone (TSH) at 24.2 µIU/mL (normal: 0.27-4.2), normal triiodothyronine (T3) at 45 ng/dL (normal: 60-180 ng/dL), and normal thyroxine (T4) at 0.45 ng/dL (normal: 0.93-1.7). Furthermore, the thyroglobulin level was notably high at 5,070.0 ng/mL (normal: 1.4-78.0), indicating active thyroid follicular cell production. The patient was referred to surgery and a thyroidectomy was performed.

In the post-operative phase, the patient showed signs of improvement, and she was subsequently started on a higher dose of levothyroxine (100 mcg) for thyroid hormone replacement therapy. Currently, she is under regular follow-up and demonstrating significant improvement in her symptoms. A detailed gross and microscopic examination was carried out on the excised thyroid gland.

**Figure 1:**
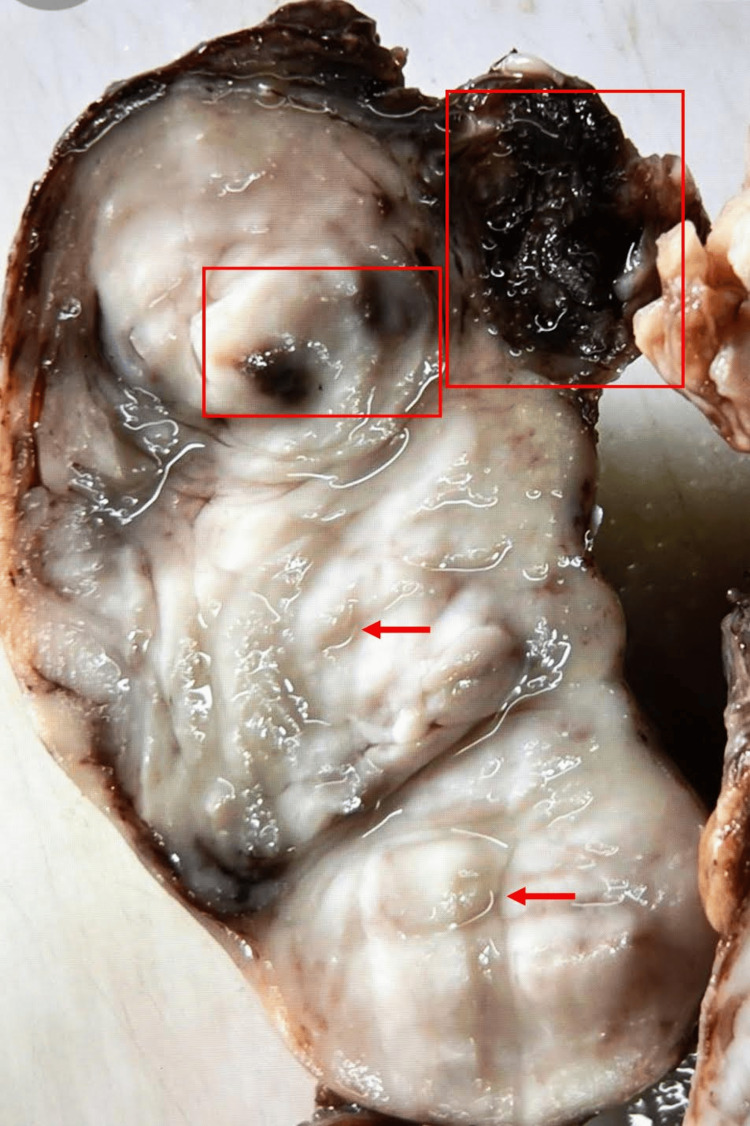
Cut section of the asymmetrically enlarged multinodular thyroid gland. The red arrows highlight firm, tan nodules of varying sizes. Hemorrhagic cyst formations are depicted within red boxes.

The gross examination of the specimen as seen in Figure 1 presented an enlarged thyroid gland with firm to rubbery texture. The gland size measured from 10.0 x 6.0 x 2.0 to 3.8 cm, demonstrating significant thyroidal pathology. The surface exhibited smooth and nodular features, pointing towards a potential existence of hyperplastic or neoplastic processes within the gland. Cut sections of the gland revealed a dull grey-tan nodular appearance with few cystic areas containing hemorrhagic fluid in line with structural changes typically seen in DG.

**Figure 2:**
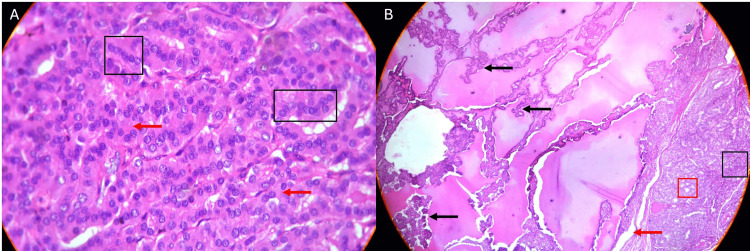
(A) Hypercellularity seen as small, compact, tightly spaced follicles (black box) with absent to minimal colloid, confirmed by the presence of empty follicles (red arrows). Nuclei are uniform with moderate hyperchromasia, and no intranuclear inclusions were observed. (B) Microphotograph two adjacent nodules. Left nodules shows prominent papillary hyperplasia (marked with black arrows). Right nodule shows microfollicular (red box) and solid growth pattern (black box). Additionally, scanty internodular tissue with fibrosis was also identified (marked with a red arrow).

Microscopic examinations were carried out on the Olympus CH2 with hematoxylin and eosin stain at a 4x magnification. Benign thyroid parenchyma was identified with partially confluent follicular nodules of varying sizes and architectural patterns, as seen in Figure 2A. These nodules demonstrated marked hyperplasia containing follicles lined by cuboidal follicular epithelium and were devoid of cytologic atypia which is a distinguishing feature of malignancy. Figure 2B shows follicles that appeared in microfollicular configurations. Only about ten percent of the follicles revealed scant, dense, luminal colloid combined with the presence of areas of prominent papillary epithelial hyperplasia. This strengthened the notion of benign thyroid enlargement. The inter-nodular tissue was scarce, and prominent fibrosis was noted alongside congested blood vessels. Importantly, no evidence of malignancy was detected in the studied sections. Hence, the patient's clinical condition could be successfully differentiated from thyroid malignancies that may present with similar clinical features but require markedly different management approaches.

## Discussion

CH represents the most prevalent metabolic disorder in newborns. If not addressed, it can cause significant neurodevelopmental defects and infertility [7]. The term DG refers to a group of congenital metabolic disorders characterized by abnormalities in thyroid hormone production. The primary causes of dyshormonogenesis include impaired organification of iodide and faulty secretion of thyroglobulin. These disruptions in thyroid hormone diminish the negative feedback directed toward the pituitary gland leading to excessive TSH production that continuously stimulates thyroid follicular cells.

While severe manifestations include neonatal or CH accompanied by goiter, mental retardation, and growth abnormalities (cretinism), less severe defects might appear later in life as goiter with minimal thyroid dysfunction [3]. Initially, the goiter presents as diffuse but progressively transforms into a nodular form [8].

The enlarged gland varies in size and is separated by fibrous strands into numerous nodules that may be encapsulated [6,8]. Although, the nodules feature a tan-yellow discoloration, the tissue between these nodules appears to be grey-brown with evidence of fibrosis, cystic changes, hemorrhaging, and minimal calcifications [8]. The nodules’ texture ranges from spongy to solid containing parts of the fragile tissue that influence the specimen’s uneven appearance [9].

Histological examination reveals a diffused distribution of cells characterized by significant follicular hyperplasia and hypercellularity [3]. The microscopic foci predominantly exhibit solid and microfollicular configurations and are often found distributed among normal tissues. The hyperplastic activity is prevalent in the entire gland leaving abnormal parenchyma between the nodules that are commonly misinterpreted as malignancy [6]. Epithelial hyperplasia characterized by varying levels of papillary proliferation with nuclear and cytologic atypia, pleomorphism, and hyperchromasia frequently occurs due to continuous TSH stimulation. In certain cases, the intensity of hyperplasia may appear to invade nearby vessel walls and capsules mimicking a pseudo-invasion [8].

The presence of severe atypia in follicular cells is characterized by the appearance of bizarre, enlarged nuclei that are either hyperchromatic or vesicular. Additionally, random nuclear atypia may be seen as enlarged pleomorphic nuclei with varying degrees of nuclear chromatin. Such presentations can lead to a misdiagnosis of follicular, papillary, medullary, or undifferentiated thyroid carcinoma. However, knowledge of the clinical context can assist in an accurate diagnosis [3].

Existing literature indicates that pronounced nuclear atypia coupled with a paucity of colloids could strongly point toward a diagnosis for DG [9]. Along with the pale and scanty colloid, the formation of papillae is frequently observed [8]. Despite these glands experiencing heightened TSH stimulation the colloid is often diminished or absent and the underlying cause remains unclear [3,10]. The presence of marked fibrosis around the nodules, along with minimal focal hemorrhages and calcifications are indicative of DG [6,9]. The absence of invasion into the capsules or vessels confirms the exclusion of papillary or follicular carcinoma [3].

Despite its infrequency, cases of follicular carcinoma, papillary thyroid carcinoma, or microcarcinoma have been identified in individuals diagnosed with DG. Consequently, even though thyroid carcinoma is uncommon in patients with multinodular DG, it is advisable to implement long-term surveillance including regular neck ultrasound evaluations. Addressing the condition early is essential, particularly in severe instances to prevent or mitigate intellectual disability and growth irregularities. The prognosis for those treated is generally favorable, longitudinal studies reveal that children who receive treatment achieve normal mean IQ levels, satisfactory academic performance, and exhibit minimal motor impairments [3].

Considering the rarity of DG, the importance of understanding the various facets of this condition is underscored by its potential implications in clinical practice, and it is especially important to avoid misdiagnosing as malignancy [9]. The diagnosis of DG can be suspected when the patient is from a non-endemic region, has no maternal history of consuming goitrogenic substances during pregnancy, and histological evidence indicating severe follicular hyperplasia [8].

## Conclusions

DG is a rare benign disorder that stems from deficient levels of thyroid hormone production and the severity of clinical manifestations can range from mild to severe. DG is characterized by a significant decrease in colloid and differs from endemic nodular goiter which typically does not show a reduction in colloid. It also lacks the degenerative changes often observed in endemic variants and exhibits nuclear atypia. It is critical to identify this condition when there are no definitive histological signs of malignancy but a history of hypothyroidism since infancy exists. The treatment approach primarily involves conservative management with lifelong thyroxine therapy; however, surgical intervention may be necessary for cosmetic reasons or to relieve tracheal compression. Long-term follow-up and regular neck ultrasound are recommended as rare occurrences of thyroid carcinoma in a dyshormonogenetic multinodular goiter have been reported. The diagnosis of DG should always consider both the clinical history and distinct histopathological characteristics. The authors urge that DG must be considered a differential where the clinical presentation of symptoms exists since birth with no obvious common cause of hypothyroidism.
